# Thyroid nodules: Global, economic, and personal burdens

**DOI:** 10.3389/fendo.2023.1113977

**Published:** 2023-01-23

**Authors:** Nishant Uppal, Reagan Collins, Benjamin James

**Affiliations:** ^1^ Harvard Medical School, Boston, MA, United States; ^2^ Department of Medicine, Brigham and Women’s Hospital, Boston, MA, United States; ^3^ Division of Surgical Oncology, Department of Surgery, Beth Israel Deaconess Medical Center, Boston, MA, United States

**Keywords:** thyroid nodule, cost effectiveness, financial burden, healthcare policy and management, economic impact

## Abstract

Thyroid nodules have garnered attention due to changes in population surveillance systems and rising concerns about the associated financial burden on healthcare systems, payers, and patients. In this review, we find that prevalence rates vary widely based on method of detection and may particularly pronounced in asymptomatic patients undergoing routine screening. Incidence rates may be particularly rising in lower-income and middle-income countries and may be declining in higher-income countries. Despite high incidence rates, survival rates continue to be as high as 97% for papillary thyroid cancer. Over the last few decades, thyroid nodule workup and management has grown more sophisticated with the advent of fine-needle aspiration biopsy, specialized biomarkers, and molecular testing. However, gaps remain in risk stratification that can lead to substantial costs of care. Certain molecular tests, such as the Afirma Gene Sequencing Classifier can lead to a cost per diagnosis of $17,873 while achieving only mild decreases in diagnostic lobectomies for patients (11.6% to 9.7% in one study). Out-of-pocket costs associated with thyroid nodule management continue to drive significant financial toxicity for patients, especially for individuals with thyroid cancer. Financial toxicity has been defined as a term that describes how direct and indirect medical costs of cancer care strain patients and households *via* decreased income, assets, and spending on basic necessities. Recent studies suggest that such toxicity can lead to adverse financial outcomes, such as foreclosure and bankruptcy. Additional cost-effectiveness analyses are needed to improve existing thyroid nodule management systems and new clinical tools are needed to avoid unnecessary workup and management.

## Introduction

Increasing rates of thyroid nodule detection has prompted assessment of the global, economic, and patient-borne burden associated with the evaluation and treatment of benign and malignant disease. The global burden of thyroid nodules reflects differences in population surveillance across countries that has led to variation in thyroid nodule incidence rates. For payers, disparate systems for covering healthcare costs yields unique economic considerations associated with financing the costs of thyroid nodule workup and management. High out-of-pocket costs also lead to patient concerns with managing diagnostic workup and treatment of thyroid nodules. Here, we describe what is known about the global, economic, and patient-borne burdens of thyroid nodule management and outline strategies for mitigating the societal and financial implications associated with potentially unnecessary or extraneous care.

## Global burden of thyroid nodules

The prevalence of thyroid nodules among the general population has been estimated as upwards of 67% depending on mode of detection (palpation, ultrasound or autopsy) and varies widely by country (1–3). Prevalence ranges between 34% to 66% depending on ultrasound detection rates or autopsy findings (2, 4). Female sex, higher body mass index (BMI), and older age are associated with an increased prevalence of thyroid nodules (5, 6). Although high prevalence rates suggest a significant burden of disease, most thyroid nodules are benign or have no ultrasound features to suggest malignancy and thus are largely clinically insignificant (7, 8). When evaluating mechanism of nodule diagnosis on a global scale, Sajisevi et al. found variation across participating countries (9). Rates of nodule diagnosis secondary to symptomatic presentation were much higher in South Africa and Denmark at 79% and 54%, respectively, while rates were similar and much lower in the United States and Canada at around 30% (9). However, thyroid surgery was performed more often in asymptomatic patients in the United States and Canada which has substantial impact when considering the effective management of nodules without overtreatment (9).

The widespread adoption of sensitive imaging techniques has contributed to the increasing frequency of detection of incidental thyroid nodules (10). Due to the relatively indolent nature of thyroid nodules, the primary clinical concern is excluding malignancy. Thus, the complex diagnostic assessment of thyroid nodules largely pertains to determining clinical significance while avoiding overdiagnosis and overtreatment (11, 12). Of detected nodules, 10-15% represent malignant disease (11). Globally, incidence rates of thyroid cancer have grown substantially with the adoption of widespread thyroid ultrasound use, which has raised concerns of the overdiagnosis of subclinical thyroid disease. In the United States, the incidence of thyroid cancer tripled from 1975 to 2009 (13), which resembles trends in other countries, such as South Korea (14, 15). Increases are largely due to increasing detection of low-risk subclinical papillary thyroid microcarcinoma (14). Survival rates have remained as high as 97% for papillary thyroid cancer, the most common type of thyroid cancer (16). From 1978 to 2007, mortality rates steadily declined in most countries with reported mortality rate reductions of 43.2% for men, and 50% for women (17). Further, men in China and women in Australia were noted to have the largest decreases in mortality rates during this time (17). Consideration of the falling mortality rates despite the rising incidence further supports the concern for overdiagnosis and overtreatment of thyroid cancer on a global scale (16).

The trend of rising incidence began to regress first in South Korea in 2014 with a 30% reduction in nodule detection in response to less screening, and as a result, less diagnosis (15). Similarly, recent findings from the Global Burden of Disease Study found that incidence rates have started to plateau in EU15+ nations and in the US between 2011 and 2019 (18). However, in low- and middle-income countries, incidence rates have continued to rise (19). Although the incidence in high-income countries may be decreasing, overall rates of thyroid cancer are still highest in these countries with the most incident cases in China, the United States, and India (18, 20). Further, significant variability in reported rates exists globally. For instance, there is a fivefold difference in thyroid cancer incidence rates in women across various regions of the world (17). Despite the regional variation in incidence by sex, the observed female to male ratio is relatively consistent across all regions at 3:1 (17). Possible contributing factors to the regional variation include barriers to access to health care, higher levels of radiation exposure, and iodine deficiency present in certain low- and middle-income countries (20, 21). This suggests that although over screening and overdiagnosis could be contributing to the high incidence in high-income countries, the variability in other parts of the world may be a true rise in incidence due to environmental exposures or modifiable risk factors. However, recent work on US trends in thyroid cancer mortality has suggested that incidence-based mortality may be growing by as much as 1.1% annually for all thyroid cancer patients and 2.9% for advanced-stage papillary thyroid carcinoma (22). Therefore, robustly characterizing the burden of thyroid nodules may require additional research on thyroid cancer incidence and mortality that accounts for demographic and tumor characteristics.

## Economic burden of thyroid nodule care

While the global burden of disease has been frequently reported, the economic burden associated with thyroid nodules is only partially understood. For nodules representing malignant disease, the costs of well-differentiated thyroid cancer care in the United States are projected to exceed $3.5 billion by 2030 (23). The plurality (41%) of healthcare expenditures is incurred for newly diagnosed patients. Initial diagnosis and evaluation, including primary care provider visit, endocrinology/surgical consultations, ultrasound imaging, and fine-needle aspiration biopsy (FNA) drive the economic burden of thyroid nodule management for both benign and malignant disease. American Thyroid Association (ATA) guidelines suggest that FNA is the most cost-effective method for evaluating thyroid nodules and further recommends ultrasound guidance, which has been shown to achieve better diagnostic accuracy than palpation alone (24–26). For incidental thyroid nodules that are less than 2 centimeters, the cost-effectiveness of FNA appears poor compared to observation ($542 vs. $412 in direct costs) (27).

Prior cost-effectiveness analyses have estimated that the screening and management of all thyroid nodules in the United States would incur $25.1 billion in costs, and the addition of specialized biomarkers, such as serum calcitonin for medullary thyroid cancer, to current ATA guidelines could add $1.4 billion in costs, which would represent a mean $11,793 per life-year saved (28). Rather than routine use of adjunct testing, other studies of the cost of thyroid nodule evaluation considered adding molecular testing only for individuals with indeterminate thyroid nodules based on initial FNA cytology results (29), which represent 20-30% of FNA results. Yip et al. found that while molecular testing added $104 per patient to the costs of thyroid nodule diagnostic workup, cost savings were realized by a decrease in the proportion of diagnostic lobectomies compared to standard care (11.6% to 9.7%) (29). The Afirma Gene Sequencing Classifier™ and ThyroSeq™ are two nucleic-acid based molecular tests that use gene expression profiling and/or genotyping of tumor-associated genetic mutations to attempt to determine the likelihood that samples represent malignancy (28–31). The cost-effectiveness of molecular testing also varies between Afirma, which may be more costly than lobectomy (30), and ThyroSeq v3, which was shown in a single-center Canadian study to reduce the number of diagnostic lobectomies (31). A comparative study of both molecular testing options suggested that for indeterminate nodules, both Afirma and ThyroSeq v3 were more cost-effective than lobectomy, but ThyroSeq v3 yielded a cost per diagnosis of $14,277 compared to $17,873 for the Afirma Gene Sequencing Classifier (32). Molecular tests may be used more often in the United States than in other countries. However, due to the relatively recent emergence and evolution of molecular testing, the particular extent to which the use of such tests varies between countries has not yet been fully characterized.

Although not routinely performed, intraoperative frozen section analysis can also be a driver of the economic burden of thyroid nodule management and includes potential costs from testing, labor, extended operating room time, and completion thyroidectomies in some cases (33, 34). One meta-analysis suggested that frozen section analysis offered only moderate diagnostic utility (sensitivity, 95% CI: 43%, 34%-53%) and routine use should be discouraged for follicular neoplasms (34). A separate cost analysis instead suggested that routine use of frozen section for patients with “suspicious for malignancy” cytology during thyroid lobectomy could actually achieve costs of $474 per case, primarily due to a large reduction in rates of subsequent total thyroidectomy compared to standard care (7.7% vs. 26.1%) (35).

## Patient-borne financial burden of thyroid nodule management

Finally, the patient-borne financial burden of thyroid disease has been assessed using both out-of-pocket costs and perceived financial toxicity as primary metrics. Out-of-pocket costs are driven by the surgical management of thyroid disease, which are substantial for both benign and malignant conditions and pronounced even for commercial insured patients (15). However out-of-pocket costs for patients who do not undergo surgical management for thyroid nodules remain due to the diagnostic sequelae of incidental detection, including active surveillance which includes lab testing and recurrent imaging. Patients who self-identify with overdiagnosed thyroid cancer but opt for nonintervention are at risk for healthcare disengagement and lower quality-of-life (36). Current estimates of the perceived financial burden rely primarily on cohort and cross-sectional studies of thyroid cancer patients, which have shown that 46.1% of patients endorse a psychological financial burden and 28.1% of patients endorse a material financial burden (37). There is also evidence of household strain associated with thyroid cancer diagnosis and treatment on patients with 48% patients reporting reduced income, 9% losing insurance coverage as well as 18.1% reporting unemployment for at least 6 months (38, 39). Thyroid cancer care has also been associated with adverse financial outcomes, including a higher likelihood of notice of default and foreclosure and bankruptcy compared to other cancer types (40, 41). Notably, bankruptcy rates have been estimated to be as high as 41% at 5 years after diagnosis despite high survival rates (98% at 5 years after diagnosis) (42). Previously, we have summarized the financial burden of thyroid cancer and outlined frameworks for improving research designed to measure and mitigate the financial burden of care (43).

Evidence further suggests that overdiagnosis and overtreatment of thyroid cancer can also impair health-related quality of life (HRQoL) for patients. Thyroid cancer survivors cite declines in psychological and emotional well-being due to anxiety and depression associated with treatment, and these symptoms may persist during remission because patients often fear recurrence of cancer (44). For patients who have undergone thyroidectomy, surveillance costs can also contribute to reduced quality of life and excess out-of-pocket spending, especially since the cost to detect 1 recurrence has been estimated at $147,819 (45, 46). As we have summarized previously, the costs of thyroid cancer diagnosis and treatment lead many patients to delay care and may risk spending on other medical conditions that contribute substantially to improved health, quality of life, and lifespan (43).

## Discussion

Recent retrospective analyses have found that 41% of patients undergoing surgical treatment for thyroid nodules have no thyroid-referable symptoms at the time of detection, and the mean tumor size is smaller in asymptomatic patients (2.1 cm) compared to symptomatic patients (3.2 cm) (9). An additional meta-analysis showed that 68.8% of all thyroid nodules undergoing surgical excision represented benign disease (47). This suggests that increasing detection of benign and subclinical disease may be generating excess healthcare costs. The thyroid nodule diagnostic workup routinely involves ultrasound imaging and FNA for nodules considered suspicious for malignancy based on sonographic features. In the United States, the American College of Radiology Thyroid Imaging Reporting and Data System (TIRADS) risk stratification system is used to guide subsequent management of thyroid nodules undergoing sonographic evaluation (48). This points system creates categories and biopsy thresholds for consideration of FNA based on risk of malignancy but does not additionally incorporate cost-effectiveness estimates nor stratifies by thyroid cancer subtype. The latter is particularly important considering the significant differences in 5-year relative survival rates between patients with follicular and medullary thyroid cancer compared to papillary thyroid cancer (49). Thus, there are likely many patients with borderline radiographic features (i.e. TR2 and TR3 classifications) who still undergo unnecessary FNA despite low malignancy risk and potentially little benefit from earlier detection of indolent follicular thyroid cancer types. In an analogous fashion, the Bethesda classification system for thyroid cytopathology may lead to unnecessary thyroid surgery in many patients with indeterminate typical findings from FNA (50), and there remains debate as to whether molecular testing substantially reduces the costs of care for indeterminate nodules given the high costs of Afirma and ThyroSeq v3 testing. One potential driver is clinical concern that the risk of malignancy for pathologically analyzed samples is underestimated (50). Although thyroid cancer incidence rates have started to plateau in the US after changes in ATA recommendations, disability-adjusted life years have not yet improved which may reflect suboptimal risk stratification and higher average healthcare expenditures relative to other countries (18).

The high costs of care in the United States imply a different risk calculus for assessing the risks and benefits of thyroid cancer care, especially since patients bear substantial out-of-pocket costs for diagnostic workup, surgical management, and surveillance. Importantly, thyroid cancer patients remain at risk for adverse financial outcomes and material and psychological hardship that could impair quality of life more than certain untreated forms of thyroid cancer, such as papillary thyroid microcarcinoma, that are unlikely to produce symptoms or metastasize. Furthermore, these risks do not appear to abate during remission, as patients continue to incur costs due to surveillance and experience burden associated with the fear of recurrence. Therefore, thyroid nodule management may need to be tailored in the United States to the unique healthcare system reimbursement structure and high patient-borne costs of care compared to countries with single-payer systems or alternative payment schemes in which patients pay a smaller proportion of household income towards medical care.

## Author contributions

All authors listed have made a substantial, direct, and intellectual contribution to the work and approved it for publication.

## References

[1] Tamhane S Gharib H. Thyroid nodule update on diagnosis and management. Clin Diabetes Endocrinol (2016) 2:17. doi: 10.1186/s40842-016-0035-7 28702251 PMC5471878

[2] Li Y Jin C Li J Tong M Wang M Huang J. Prevalence of thyroid nodules in China: A health examination cohort-based study. Front Endocrinol (2021) 12:676144. doi: 10.3389/fendo.2021.676144 PMC8188053 34122350

[3] Tan GH Gharib H. Thyroid incidentalomas: management approaches to nonpalpable nodules discovered incidentally on thyroid imaging. Ann Intern Med (1997) 126(3):226–31. doi: 10.7326/0003-4819-126-3-199702010-00009 9027275

[4] Sosa JA Hanna JW Robinson KA Lanman RB. Increases in thyroid nodule fine-needle aspirations, operations, and diagnoses of thyroid cancer in the united states. Surgery (2013) 154(6):1420–6; discussion 1426–7. doi: 10.1016/j.surg.2013.07.006 24094448

[5] Kwong N Medici M Angell TE Liu X Marqusee E Cibas ES. The influence of patient age on thyroid nodule formation, multinodularity, and thyroid cancer risk. J Clin Endocrinol Metab (2015) 100(12):4434–40. doi: 10.1210/jc.2015-3100 PMC4667162 26465395

[6] Guth S Theune U Aberle J Galach A Bamberger CM. Very high prevalence of thyroid nodules detected by high frequency (13 MHz) ultrasound examination. Eur J Clin Invest. (2009) 39(8):699–706. doi: 10.1111/j.1365-2362.2009.02162.x 19601965

[7] Durante C Grani G Lamartina L Filetti S Mandel SJ Cooper DS. The diagnosis and management of thyroid nodules: A review. JAMA (2018) 319(9):914–24. doi: 10.1001/jama.2018.0898 29509871

[8] Filetti S Durante C Torlontano M. Nonsurgical approaches to the management of thyroid nodules. Nat Clin Pract Endocrinol Metab (2006) 2(7):384–94. doi: 10.1038/ncpendmet0215 16932321

[9] Sajisevi M Caulley L Eskander A Du YJ Auh E Karabachev A. Evaluating the rising incidence of thyroid cancer and thyroid nodule detection modes: A multinational, multi-institutional analysis. JAMA Otolaryngol Head Neck Surg (2022) 14:811–8. doi: 10.1001/jamaoto.2022.1743 PMC9284406 35834240

[10] Dean DS Gharib H. Epidemiology of thyroid nodules. Best Pract Res Clin Endocrinol Metab (2008) 22(6):901–11. doi: 10.1016/j.beem.2008.09.019 19041821

[11] Alexander EK Cibas ES. Diagnosis of thyroid nodules. Lancet Diabetes Endocrinol (2022) 10(7):533–9. doi: 10.1016/S2213-8587(22)00101-2 35752200

[12] Alexander EK Doherty GM Barletta JA. Management of thyroid nodules. Lancet Diabetes Endocrinol (2022) 10(7):540–8. doi: 10.1016/S2213-8587(22)00139-5 35752201

[13] Davies L Welch HG. Current thyroid cancer trends in the united states. JAMA Otolaryngol Head Neck Surg (2014) 140(4):317–22. doi: 10.1001/jamaoto.2014.1 24557566

[14] Powers AE Marcadis AR Lee M Morris LGT Marti JL. Changes in trends in thyroid cancer incidence in the united states, 1992 to 2016. JAMA (2019) 322(24):2440–1. doi: 10.1001/jama.2019.18528 PMC6990659 31860035

[15] Ahn HS Welch HG. South korea’s thyroid-cancer “Epidemic”–turning the tide. N Engl J Med (2015) 373(24):2389–90. doi: 10.1056/NEJMc1507622 26650173

[16] Aschebrook-Kilfoy B James B Nagar S Kaplan S Seng V Ahsan H. Risk factors for decreased quality of life in thyroid cancer survivors: Initial findings from the north American thyroid cancer survivorship study. Thyroid (2015) 25(12):1313–21. doi: 10.1089/thy.2015.0098 PMC4684649 26431811

[17] James BC Mitchell JM Jeon HD Vasilottos N Grogan RH Aschebrook-Kilfoy B. An update in international trends in incidence rates of thyroid cancer, 1973-2007. Cancer Causes Control. (2018) 29(4-5):465–73. doi: 10.1007/s10552-018-1023-2 29623496

[18] Schuster-Bruce J Jani C Goodall R Kim D Hughes W Salciccioli JD. A comparison of the burden of thyroid cancer among the European union 15+ countries, 1990-2019: Estimates from the global burden of disease study. JAMA Otolaryngol Head Neck Surg (2022) 148(4):350–9. doi: 10.1001/jamaoto.2021.4549 PMC8914910 35266977

[19] Deng Y Li H Wang M Li N Tian T Wu Y. Global burden of thyroid cancer from 1990 to 2017. JAMA Netw Open (2020) 3(6):e208759. doi: 10.1001/jamanetworkopen.2020.8759 32589231 PMC7320301

[20] Kim J Gosnell JE Roman SA. Geographic influences in the global rise of thyroid cancer. Nat Rev Endocrinol (2020) 16(1):17–29. doi: 10.1038/s41574-019-0263-x 31616074

[21] Wiltshire JJ Drake TM Uttley L Balasubramanian SP. Systematic review of trends in the incidence rates of thyroid cancer. Thyroid (2016) 26(11):1541–52. doi: 10.1089/thy.2016.0100 27571228

[22] Lim H Devesa SS Sosa JA Check D Kitahara CM. Trends in thyroid cancer incidence and mortality in the united states, 1974-2013. JAMA (2017) 317(13):1338–48. doi: 10.1001/jama.2017.2719 PMC8216772 28362912

[23] Lubitz CC Kong CY McMahon PM Daniels GH Chen Y Economopoulos KP. Annual financial impact of well-differentiated thyroid cancer care in the united states. Cancer (2014) 120(9):1345–52. doi: 10.1002/cncr.28562 PMC3999178 24481684

[24] Haugen BR Alexander EK Bible KC Doherty GM Mandel SJ Nikiforov YE. 2015 American Thyroid association management guidelines for adult patients with thyroid nodules and differentiated thyroid cancer: The American thyroid association guidelines task force on thyroid nodules and differentiated thyroid cancer. Thyroid (2016) 26(1):1–133. doi: 10.1089/thy.2015.0020 26462967 PMC4739132

[25] Danese D Sciacchitano S Farsetti A Andreoli M Pontecorvi A. Diagnostic accuracy of conventional versus sonography-guided fine-needle aspiration biopsy of thyroid nodules. Thyroid (1998) 8(1):15–21. doi: 10.1089/thy.1998.8.15 9492148

[26] Carmeci C Jeffrey RB McDougall IR Nowels KW Weigel RJ. Ultrasound-guided fine-needle aspiration biopsy of thyroid masses. Thyroid (1998) 8(4):283–9. doi: 10.1089/thy.1998.8.283 9588492

[27] Wong CKH Liu X Lang BHH. Cost-effectiveness of fine-needle aspiration cytology (FNAC) and watchful observation for incidental thyroid nodules. J Endocrinol Invest. (2020) 43(11):1645–54. doi: 10.1007/s40618-020-01254-0 32307641

[28] Cheung K Roman SA Wang TS Walker HD Sosa JA. Calcitonin measurement in the evaluation of thyroid nodules in the united states: a cost-effectiveness and decision analysis. J Clin Endocrinol Metab (2008) 93(6):2173–80. doi: 10.1210/jc.2007-2496 18364376

[29] Yip L Farris C Kabaker AS Hodak SP Nikiforova MN McCoy KL. Cost impact of molecular testing for indeterminate thyroid nodule fine-needle aspiration biopsies. J Clin Endocrinol Metab (2012) 97(6):1905–12. doi: 10.1210/jc.2011-3048 PMC3791417 22419727

[30] Balentine CJ Vanness DJ Schneider DF. Cost-effectiveness of lobectomy versus genetic testing (Afirma®) for indeterminate thyroid nodules: Considering the costs of surveillance. Surgery (2018) 163(1):88–96. doi: 10.1016/j.surg.2017.10.004 29128178 PMC5736452

[31] Chen T Gilfix BM Rivera J Sadeghi N Richardson K Hier MP. The role of the ThyroSeq v3 molecular test in the surgical management of thyroid nodules in the Canadian public health care setting. Thyroid (2020) 30(9):1280–7. doi: 10.1089/thy.2019.0539 32242511

[32] Nicholson KJ Roberts MS McCoy KL Carty SE Yip L. Molecular testing versus diagnostic lobectomy in Bethesda III/IV thyroid nodules: A cost-effectiveness analysis. Thyroid (2019) 29(9):1237–43. doi: 10.1089/thy.2018.0779 PMC7366255 31407625

[33] Alci E Makay Ö. Impact of healthcare resources on management of indeterminate thyroid tumors. Ann Thyroid (2021) 6:3–3. doi: 10.21037/aot-20-44

[34] Grisales J Sanabria A. Utility of routine frozen section of thyroid nodules classified as follicular neoplasm. Am J Clin Pathol (2020) 153(2):210–20. doi: 10.1093/ajcp/aqz152 31732728

[35] Bollig CA Gilley D Lesko D Jorgensen JB Galloway TL Zitsch RP 3rd. Economic impact of frozen section for thyroid nodules with “Suspicious for malignancy” cytology. Otolaryngol Head Neck Surg (2018) 158(2):257–64. doi: 10.1177/0194599817740328 29292662

[36] Davies L Hendrickson CD Hanson GS. Experience of US patients who self-identify as having an overdiagnosed thyroid cancer: A qualitative analysis. JAMA Otolaryngol Head Neck Surg (2017) 143(7):663–9. doi: 10.1001/jamaoto.2016.4749 PMC5824211 28278335

[37] Barrows CE Belle JM Fleishman A Lubitz CC James BC. Financial burden of thyroid cancer in the united states: An estimate of economic and psychological hardship among thyroid cancer survivors. Surgery (2020) 167(2):378–84. doi: 10.1016/j.surg.2019.09.010 31653488

[38] Broekhuis JM Li C Chen HW Chaves N Duncan S Lopez B. Patient-reported financial burden in thyroid cancer. J Surg Res (2021) 266:160–7. doi: 10.1016/j.jss.2021.03.051 34000639

[39] Mongelli MN Giri S Peipert BJ Helenowski IB Yount SE Sturgeon C. Financial burden and quality of life among thyroid cancer survivors. Surgery (2020) 167(3):631–7. doi: 10.1016/j.surg.2019.11.014 31862171

[40] Gupta A Morrison ER Fedorenko C Ramsey S. Leverage, default, and mortality: Evidence from cancer diagnoses (2017). Available at: https://papers.ssrn.com/abstract=2583975.

[41] Ramsey S Blough D Kirchhoff A Kreizenbeck K Fedorenko C Snell K. Washington State cancer patients found to be at greater risk for bankruptcy than people without a cancer diagnosis. Health Aff . (2013) 32(6):1143–52. doi: 10.1377/hlthaff.2012.1263 PMC4240626 23676531

[42] Ramsey SD Bansal A Fedorenko CR Blough DK Overstreet KA Shankaran V. Financial insolvency as a risk factor for early mortality among patients with cancer. J Clin Oncol (2016) 34(9):980–6. doi: 10.1200/JCO.2015.64.6620 PMC4933128 26811521

[43] Uppal N Cunningham Nee Lubitz C James B. The cost and financial burden of thyroid cancer on patients in the US: A review and directions for future research. JAMA Otolaryngol Head Neck Surg (2022) 148(6):568–75. doi: 10.1001/jamaoto.2022.0660 35511135

[44] Roth EM Lubitz CC Swan JS James BC. Patient-reported quality-of-Life outcome measures in the thyroid cancer population. Thyroid (2020) 30(10):1414–31. doi: 10.1089/thy.2020.0038 PMC7583324 32292128

[45] Wang LY Roman BR Migliacci JC Palmer FL Tuttle RM Shaha AR. Cost-effectiveness analysis of papillary thyroid cancer surveillance. Cancer (2015) 121(23):4132–40. doi: 10.1002/cncr.29633 PMC4976498 26280253

[46] Wu JX Beni CE Zanocco KA Sturgeon C Yeh MW. Cost-effectiveness of long-term every three-year versus annual postoperative surveillance for low-risk papillary thyroid cancer. Thyroid (2015) 25(7):797–803. doi: 10.1089/thy.2014.0617 25851702

[47] Bongiovanni M Spitale A Faquin WC Mazzucchelli L Baloch ZW. The Bethesda system for reporting thyroid cytopathology: a meta-analysis. Acta Cytol. (2012) 56(4):333–9. doi: 10.1159/000339959 22846422

[48] Middleton WD Teefey SA Reading CC Langer JE Beland MD Szabunio MM. Multiinstitutional analysis of thyroid nodule risk stratification using the American college of radiology thyroid imaging reporting and data system. AJR Am J Roentgenol. (2017) 208(6):1331–41. doi: 10.2214/AJR.16.17613 28402167

[49] Survival rates for thyroid cancer (2022). Available at: https://www.cancer.org/cancer/thyroid-cancer/detection-diagnosis-staging/survival-rates.html.

[50] Cibas ES Ali SZ. The 2017 Bethesda system for reporting thyroid cytopathology. Thyroid (2017) 27(11):1341–6. doi: 10.1089/thy.2017.0500 29091573

